# Clinical characteristics and outcomes of hospitalized patients with intracranial hemorrhage after percutaneous coronary intervention

**DOI:** 10.3389/fcvm.2025.1424598

**Published:** 2025-03-11

**Authors:** Yujing Zhou, Xin Su, Peng Liu, Yi Tang, Dong Cheng, Haiyu Li, Haiqiang Sang

**Affiliations:** Department of Cardiology, The First Affiliated Hospital of Zhengzhou University, Zhengzhou, China

**Keywords:** intracranial hemorrhage, percutaneous coronary intervention, coronary artery disease, mortality, clinical characteristics

## Abstract

**Objectives:**

Complications of intracranial hemorrhage (ICH) after percutaneous coronary intervention (PCI), although rare, have a poor prognosis with high mortality rates. This study aims to provide information on the clinical characteristics and outcomes of hospitalized patients with ICH after PCI.

**Materials and methods:**

This retrospective study included 24 patients enrolled from February 2014 to September 2023, which occurred ICH during post-PCI hospitalization. We mainly analyzed general, procedural, ICH features and subsequent outcomes. In addition, the predictive ability of the CRUSADE, ARC-HBR, and ACUITY scores was assessed with the receiver operating characteristics area under the curve (AUC).

**Results:**

Among the 24 patients, the mean age was 62.21 ± 10.01 years, and 66.7% (*n* = 16) were men. The mortality of ICH patients after PCI was very high (*n* = 13, 54.2%). In addition, the most common initial manifestation of ICH patients was the disturbance of consciousness (*n* = 14, 58.3%). Over half of the cases (58.3%) occurred ICH within the first 12 h following PCI. 13 patients (54.2%) had an ICH volume ≥30 cm^3^, and of these patients, a total of 11(84.6%) died. ICH volume ≥30 cm^3^ (*p* = 0.038), and the use of mechanical ventilators (*p* = 0.011) were significantly higher in patients who died. The AUC of CRUSADE, ARC-HBR, and ACUITY scores were 0.500, 0.619, and 0.545, respectively.

**Conclusions:**

In our study, the mortality of ICH after PCI was high. The high volume of ICH indicates a high risk of death.

## 1 Introduction

Coronary artery disease (CAD) is the leading cause of morbidity and mortality worldwide (1). Percutaneous coronary intervention (PCI) is the cornerstone of treatment for patients with acute coronary syndromes (ACS). In recent years, PCI has also been widely used in patients with chronic coronary syndromes (CCS) (2). In addition, antithrombotic therapy plays a crucial role in improving outcomes in patients who have undergone PCI (3). Over the past 30 years, using antiplatelet agents has significantly reduced thrombotic events and remains the standard of care following PCI in CAD patients (4). Although dual antiplatelet therapy (DAPT) reduced the risk of ischemic events, it also increased the rate of bleeding, which created a therapeutic dilemma for the clinician (5, 6). Therefore, identifying high-risk features associated with bleeding complications and implementing appropriate risk reduction approaches are essential.

Intracranial hemorrhage (ICH) is a rare but potentially life-threatening complication that may occur in patients undergoing PCI, which is the most severe bleeding complication and is often overlooked. For example, studies have shown that ICH can occur as a rare but severe complication in patients undergoing PCI, particularly those on dual antiplatelet therapy (DAPT), and may not always be adequately captured in routine clinical practice (7). Furthermore, ICH is internationally associated with significant morbidity and mortality (8, 9). Patients usually receive DAPT after PCI, and prior antiplatelet therapy is associated with higher mortality in patients with ICH (10, 11). Moreover, as there are few effective treatments for ICH, early identification of those at risk and effective preventive measures are essential. One study found that age >80 years, ICH volume >30 mm^3^, hematoma origin, intraventricular hemorrhage presence, and Glasgow Coma Scale score were associated with 30-day mortality after ICH (12).

Despite the severity of intracranial hemorrhage (ICH) following percutaneous coronary intervention (PCI), there is a paucity of detailed information regarding its clinical characteristics. Therefore, this study was conducted to provide a comprehensive description of the clinical features and outcomes of hospitalized patients who experienced ICH after PCI, thereby contributing to the existing body of knowledge in this area.

## 2 Patients and methods

### 2.1 Study population

Our study was a single-center, retrospective study of patients with ICH that occurred after PCI in the First Affiliated Hospital of Zhengzhou University from February 2014 to September 2023. A total number of 24 patients were finally enrolled after excluding individuals without PCI or ICH, patients with PCI after ICH, or those with missing data. In addition, patients were divided into a survival group (11 cases) and a death group (13 cases). Besides, the study received approval from the Human Research Ethics Committee of the First Affiliated Hospital of Zhengzhou University and the informed consent was obtained from each patient and the study protocol conforms to the ethical guidelines of the 1975 Declaration of Helsinki as reflected in *a priori* approval by the institution's human research committee.

### 2.2 Data collection

Clinical data were sourced from the Medical Information Recording System at the First Affiliated Hospital of Zhengzhou University. We retrospectively collected data concerning patients’ demographic information, days of hospitalization, medical history, diagnosis, admission features, examinations, clinical characteristics, PCI procedural and ICH-related characteristics, as well as medication usage (prior-, during-, or post-procedure).

### 2.3 Definitions

The ICH volume was measured using ABC/2 according to the head CT, in which A represented the largest diameter of the hematoma on axial images, B represented the largest diameter perpendicular to A on the same image slice, and C represented the number of slices in which the hematoma is seen, multiplied by the slice thickness (13). All lengths were registered in centimeters (cm) and volumes in cubic centimeters (cm^3^) (14). For intracerebral hemorrhages with an intraventricular extension, only the parenchymal component was measured by the ABC/2 (15). ICH was classified into two categories small (<30 cm3) or large (≥30 cm^3^) (12). Besides, the ventricular extension means the CT head confirmed that ICH had extended into the ventricles. The mortality rate mentioned in our study refers to the overall mortality within the study population during the observation period of our research. It includes both in-hospital and post-discharge mortality up to the end of the follow-up period.

Calculation of the CRUSADE score was based on the clinical data obtained at admission (heart rate, systolic blood pressure, hematocrit, creatinine clearance, gender, signs of chronic heart failure at presentation, history of vascular disease, and history of diabetes mellitus) (16). The CRUSADE score is categorized into five risk levels based on the patient's score: very low risk (≤20 points), low risk (21–30 points), moderate risk (31–40 points), high risk (41–50 points), and very high risk (>50 points). The ARC-HBR score consists of 14 primary criteria and 6 secondary criteria. If a patient meets at least 1 primary criterion or 2 secondary criteria, they may be considered to have a high risk of bleeding (17). The ACUITY score was based on the clinical data obtained at admission [age, gender, serum creatinine, white blood cell count, anemia, type of ACS (unstable angina, non-ST-elevation or ST-elevation acute myocardial infarction), use of bivalirudin] (18). The ACUITY score is categorized into four risk levels based on the patient's score: low risk (<10 points), moderate risk (10–14 points), high risk (15–19 points), and very high risk (≥20 points). The higher the scores, the higher the patient's risk of bleeding. In our study, all the scores of the patients were classified into bleeding risk strata by considering the very high risk and high risk as unique categories (high risk) and very low risk and low risk as low risk categories (low risk).

### 2.4 Statistical analysis

Categorical variables were shown as frequencies and percentages, whereas continuous variables were presented as mean ± standard deviations (SD). Continuous variables were compared using the one-way ANOVA analysis, whereas categorical variables were compared using Fisher's exact test. In addition, in our study, the ROC curve analysis was conducted to evaluate the predictive effectiveness of the CRUSADE, ARC-HBR, and ACUITY scores specifically for in-hospital bleeding events among patients with intracranial hemorrhage after percutaneous coronary intervention (PCI). The AUC values were calculated to assess the ability of these scores to predict the occurrence of bleeding complications during hospitalization.Statistical analyses were performed using SPSS 25.0 software. A two-sided *P*-value < 0.05 was considered statistically significant.

## 3 Results

### 3.1 General characteristics

A total of 24 patients experienced ICH after undergoing PCI between October 2015 and October 2023. Baseline characteristics are presented in Table 1. Of the 24 patients, the mean age was 62.21 ± 10.01 years, 66.7% (*n* = 16) were men, 11(45.8%) patients experienced myocardial infarction (MI), and 13 (54.2%) experienced unstable angina (UA). In addition, 15 patients (62.5%) had hypertension, and 5 patients (20.8%) had diabetes mellitus. The median follow-up time for our cohort was 11.00 days (IQR: 6.00–28.25 days). During this period, we observed an in-hospital mortality rate of 54.2% (*n* = 13).

**Table 1:** Baseline characteristics.

Clinical data	Total (*n* = 24)	Survival (*n* = 11)	Death (*n* = 13)	*P*-value
Demographic variables				
Male, *n* (%)	16 (66.7)	8 (72.7)	8 (61.5)	0.679
Age, years	62.21 ± 10.01	61.64 ± 6.83	62.69 ± 12.35	0.803
hospital stay, days	15.33 ± 11.62	25.82 ± 8.27	6.46 ± 3.99	<0.001
Medical history, *n* (%)				
Hypertension	15 (62.5)	7 (63.6)	8 (61.5)	1.000
Diabetes mellitus	5 (20.8)	2 (18.2)	3 (23.1)	1.000
Peripheral vascular disease	14 (58.3)	4 (36.4)	10 (76.9)	0.095
Heart failure	8 (33.3)	3 (27.3)	5 (38.5)	0.679
Prior ischemia stroke/TIA	6 (25.0)	2 (18.2)	4 (30.8)	0.649
Dyslipidemia	4 (16.7)	2 (18.2)	2 (15.4)	1.000
Anemia	8 (33.3)	5 (45.5)	3 (23.1)	0.390
Prior PCI	4 (16.7)	3 (27.3)	1 (7.7)	0.300
Renal insufficiency	13 (54.2)	5 (45.5)	8 (61.5)	0.682
Drinking	8 (33.3)	3 (27.3)	5 (38.5)	0.679
Smoking	10 (41.7)	6 (54.5)	4 (30.8)	0.408
Diagnosis, *n* (%)				0.444
Unstable angina	13 (54.2)	7 (63.6)	6 (46.2)	
MI	11 (45.8)	4 (36.4)	7 (53.8)	
Admission features				
SBP, mmHg	119.50 ± 23.88	114.09 ± 23.74	124.08 ± 23.96	0.318
DBP, mmHg	72.54 ± 13.11	67.36 ± 12.52	76.92 ± 12.38	0.074
LVEF, %	54.46 ± 8.45	54.73 ± 9.23	54.23 ± 8.11	0.890
LV, mm	47.75 ± 4.50	48.18 ± 4.96	47.38 ± 4.23	0.675
HR, beats/min	77.25 ± 14.39	78.81 ± 12.00	75.08 ± 16.31	0.434
Examinations				
WBC, 109/L	13.65 ± 6.13	12.29 ± 5.00	14.79 ± 6.94	0.330
RBC, 109/L	3.60 ± 0.83	3.51 ± 0.91	3.68 ± 0.79	0.620
Hemoglobin, g/dl	109.84 ± 25.38	105.84 ± 27.40	113.23 ± 24.13	0.489
Platelet, 109/L	163.61 ± 77.97	160.18 ± 82.86	166.51 ± 76.86	0.848
Cr, µmol/L	93.06 ± 54.58	95.46 ± 55.20	91.03 ± 56.22	0.848
UA, µmol/L	325.71 ± 200.08	322.64 ± 221.33	328.31 ± 189.44	0.947
eGFR, ml/min/1.73 m^2^	71.69 ± 24.34	71.87 ± 26.10	71.53 ± 23.92	0.974
Albumin, g/L	36.60 ± 5.27	35.94 ± 5.38	37.15 ± 5.33	0.584
HbA1C	6.04 ± 0.72	6.14 ± 0.91	5.96 ± 0.53	0.544
PT, s	12.18 ± 1.85	12.62 ± 1.93	11.81 ± 1.76	0.294
APTT, s	40.80 ± 32.90	32.29 ± 5.24	47.98 ± 43.93	0.253
D-dimer, mg/L	4.75 ± 5.14	3.71 ± 4.34	5.63 ± 5.76	0.374
TC, mmol/L	3.81 ± 1.19	4.14 ± 1.27	3.54 ± 1.10	0.228
TG, mmol/L	1.50 ± 0.98	1.49 ± 1.12	1.51 ± 0.88	0.969
HDL, mmol/L	1.01 ± 0.36	1.03 ± 0.40	0.99 ± 0.33	0.759
LDL-C, mmol/L	2.34 ± 1.12	2.67 ± 1.42	2.06 ± 0.74	0.191
Tn I, µg/L	4.34 ± 4.68	3.55 ± 4.06	5.01 ± 5.22	0.457
Clinical characteristics, *n* (%)				
ECMO	3 (12.5)	1 (9.1)	2 (15.4)	1.000
Mechanical ventilator	19(79.2)	6(54.5)	13(100.0)	0.011

CHD, coronary heart disease; PCI, percutaneous coronary intervention; TIA, transient ischemic attack; MI, myocardial infarction; SBP, systolic blood pressure; DBP, diastolic blood pressure; LVEF, left ventricular ejection fraction; LV, left ventricular; HR, heart rate; WBC, white blood cell; RBC, red blood cell count; Cr, creatinine; UA: uric acid; eGFR, estimated glomerular filtration rate; HbAlC, hemoglobin A1C; PT, prothrombin time; APTT, activated partial thromboplastin time; TC, total cholesterol; TG, triglycerides; HDL, high-density lipoprotein; LDL-C, low-density lipoprotein cholesterol; Tn I, troponin I; ECMO, extracorporeal membrane oxygenation.

In our study, we found the proportion of patients requiring mechanical ventilation was higher in the death group [13(100.0%) vs. 6(54.5%), *p* = 0.011]. The remaining parameters were described in Table 1.

### 3.2 Procedural characteristics

Regarding procedure information, 17 patients (70.8%) underwent elective PCI, and 19 patients (79.2%) had multivessel disease. (Elective PCI was performed for patients who had stabilized after the initial acute event and required further intervention to address underlying coronary artery disease. This approach is consistent with clinical practice where elective PCI may be appropriate for post-myocardial infarction patients who have recurrent or inducible angina before hospital discharge, and for patients who have angina and remain symptomatic despite medical treatment.) Of all the patients with an indication to receive DAPT at baseline (prior to ICH onset), the majority (54.2%) received DAPT with aspirin plus ticagrelor. Overall, 20 patients (83.3%) received unfractionated heparin during PCI, and the remaining 4 patients (16.7%) received bivalirudin. Besides, one patient received tirofiban, and four received bivalirudin after PCI (Table 2).

**Table 2:** Procedural characteristics.

Variables	Total number of cases (*n* = 24)	Survival group (*n* = 11)	Death group (*n* = 13)	*P*-value
Duration of procedure, mins	76.00 ± 30.40	87.91 ± 26.15	65.92 ± 31.00	0.077
Multivessel disease, *n* (%)	19 (79.2)	9 (81.8)	10 (76.9)	1.000
Target vessel (%)				0.718
Anterior descending artery	10 (41.7)	4 (36.4)	6 (46.2)	
Circumflex	3 (12.5)	2 (18.2)	1 (7.7)	
Right coronary artery	11 (45.8)	5 (45.5)	6 (46.2)	
Timing of PCI procedure, *n* (%)				0.386
Selective	17 (70.8)	9 (81.8)	8 (61.5)	
Emergency	7 (29.2)	2 (18.2)	5 (38.5)	
Total number of stents	1.88 ± 1.12	1.55 ± 0.820	2.15 ± 1.28	0.189
Antithrombotic therapy, *n* (%)				
Pre-procedure				0.353
Aspirin plus clopidogrel	11 (45.8)	6 (54.5)	5 (38.5)	
Aspirin plus ticagrelor	13 (54.2)	5 (45.5)	8 (61.5)	
During procedure				0.300
Unfractionated heparin	20 (83.3)	8 (72.7)	12 (92.3)	
Bivalirudin	4 (16.7)	3 (27.3)	1 (7.7)	
Post-procedure				0.518
Tirofiban	1 (4.2)	1 (9.1)	0 (0.0)	
Bivalirudin	4(16.7)	2(18.2)	2(15.4)	

PCI, percutaneous coronary intervention.

### 3.3 ICH characteristics

The clinical and imaging characteristics of 24 patients with ICH are represented in Table 3. The most common initial manifestation of ICH patients was the disturbance of consciousness (*n* = 14, 58.3%), followed by focal neurological signs (*n* = 10, 41.7%). More than half of the cases (58.3%) occurred ICH within the first 12 h following PCI. All 24 patients received brain CT scans. The mean ICH volume was 40.41 ± 32.28 cm^3^, and 10 of the 13(76.9%) patients who died in the hospital had ICH volumes on CT exceeding 30 cm^3^, whereas 7 of 11(63.6%) surviving patients were discharged with cerebral hemorrhage volumes below 30 cm^3^. Out of the 9 patients with ICH who suffered ventricular extension, 6 (66.7%) died. In addition, the most common treatment of ICH patients was conservative medicine (*n* = 21, 87.5%), followed by invasive surgery (*n* = 3, 12.5%) (Table 3).

**Table 3:** ICH-related characteristics (*n* = 24).

Variables	Total number of cases (*n* = 24)	Survival group (*n* = 11)	Death group (*n* = 13)	*P*-value
Initial symptoms, *n* (%)				0.408
Focal neurological signs	10 (41.7)	6 (54.5)	4 (30.8)	
Disturbance of consciousness	14 (58.3)	5 (45.5)	9 (69.2)	
Time to symptoms after procedure				
Median time, hours	26.48 ± 41.91	35.82 ± 45.10	18.58 ± 39.03	0.326
Within 12 h, *n* (%)	14 (58.3)	5 (45.5)	9 (69.2)	0.408
More than 12 h, *n* (%)	10 (41.7)	6 (54.5)	4 (30.8)	0.408
ICH volume, cm^3^	40.41 ± 32.28	21.08 ± 16.20	56.77 ± 33.85	0.004
Small (<30)	11 (45.8)	8 (72.7)	3 (23.1)	0.038
Large (≥30)	13 (54.2)	3 (27.3)	10 (76.9)	0.038
Treatment, *n* (%)				0.576
Conservative medicine	21 (87.5)	9 (81.8)	12 (92.3)	
Minimally invasive surgery	3 (12.5)	2(18.2)	1(7.7)	

ICH, intracranial hemorrhage.

**Figure 1:**
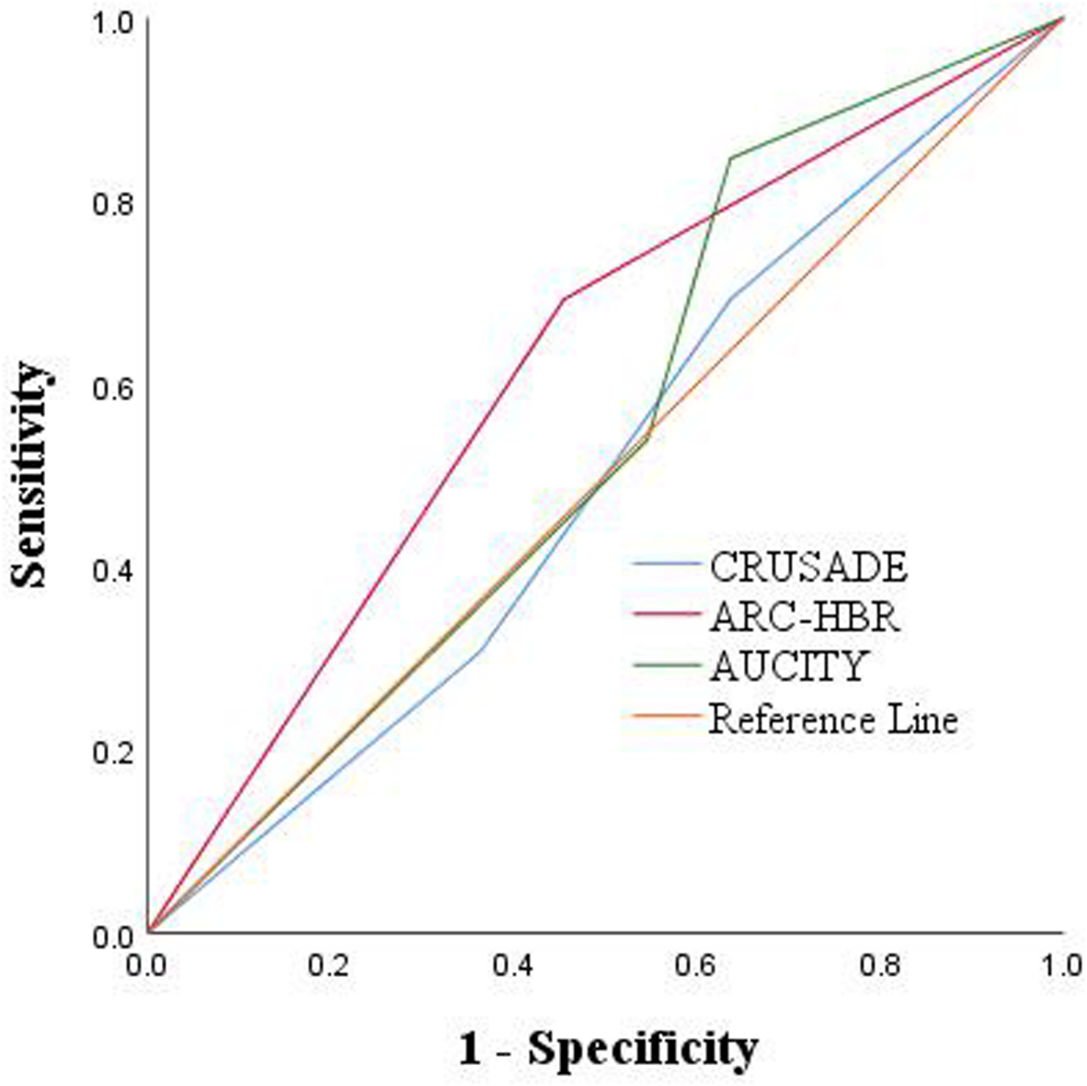
ROC: predictive outcomes with the 3 risk scores.

Among the 24 patients, 8 patients (33.3%) were classified as high or very high risk of bleeding on admission according to the CRUSADE score, 14 patients (58.3%) were classified as high risk according to the ARC-HBR score, and13 patients (54.2%) were classified as high or very high risk according to the ACUITY score. Furthermore, according to the ARC-HBR score, the mortality rate among patients with high bleeding risk was higher at 64.3% as compared to non-high bleeding risk patients, whose mortality rate was 40.0% (Table 4). Besides, ROC curve analysis was conducted to determine the AUC to judge the predictive effectiveness of CRUSADE, ARC-HBR, and ACUITY scores, with their AUC were 0.500, 0.619, and 0.545, respectively (Figure 1; Table 5).

**Table 4:** Detailed information of the 24 post-PCI patients who suffered ICH.

ID	Death	Age	Gender	Dual antiplatelet therapy, aspirin plus	During procedural anti-coagulants	Time since PCI, hours	Onset symptoms	CT Manifestations		Score			Mechanical ventilator
								Bleeding location	Volume, cm^3^	CRUSADE score	ARC-HBR score	ACUITY score	
1	YES	55	Female	Clopidogre	UFH	12.5	FNS, V	CH	20.5	Medium	Low	Medium	YES
2	YES	78	Male	Clopidogre	UFH	144	DC, FNS	FL, TL, V	50	Low	High	High	YES
3	YES	56	Male	Ticagrelor	UFH	8	DC, V	BS, V	38.2	Low	High	Medium	YES
4	NO	55	Male	Clopidogrel	UFH	65	HA	V	82	Very low	Low	High	NO
5	NO	58	Male	Ticagrelor	UFH	15	HA	TL	1	Medium	High	Very high	YES
6	YES	55	Male	Ticagrelor	Bivalirudin	2	DC, V	PL, OL, SS, CH, V	82.25	Medium	Low	Medium	YES
7	NO	55	Female	Ticagrelor	UFH	5.5	DC, HA, V	FL	41.4	High	Low	Very high	YES
8	NO	66	Female	Clopidogre	UFH	15	DC, FNS	BG, V	26	Very high	Low	Very high	YES
9	YES	55	Male	Clopidogre	UFH	29	DC, FNS	TL, SS	58.5	Medium	Low	Low	YES
10	NO	69	Female	Clopidogre	UFH	144	FNS	CE, V	46	Medium	Low	Medium	N0
11	YES	66	Male	Ticagrelor	UFH	30	DC, HA	PL, TL, OL, SS	46.25	Medium	Low	Medium	YES
12	NO	62	Male	Clopidogrel	UFH	9	V	CE, SS	8.1	Low	High	Low	N0
13	NO	69	Male	Clopidogrel	Bivalirudin	24	FNS	TL, SS	40	Low	Low	Low	NO
14	NO	50	Male	Clopidogre	UFH	90	HA, FNS	OL	3.2	Very low	High	Low	NOS
15	NO	66	Male	Ticagrelor	UFH	11.5	DC	TL, SS, V	15.2	Very high	High	Very high	YES
16	YES	72	Female	Ticagrelor	UFH	0.5		PL, TL, OL, SS	50	Medium	High	Very high	YES
17	YES	66	Female	Ticagrelor	UFH	0.5	DC	SS, CH, V	121.5	Very high	High	Very high	YES
18	YES	32	Male	Ticagrelor	UFH	5	DC	SS	3.8	Very high	High	Very high	YES
19	NO	58	Male	Clopidogrel	UFH	10	DC	TL	14	Medium	Low	Low	YES
20	YES	73	Female	Clopidogrel	UFH	2	DC	PL	55	Low	High	High	YES
21	YES	64	Female	Ticagrelor	UFH	1	DC	TL, SS, V	108	Very high	High	High	YES
22	NO	70	Male	Ticagrelor	UFH	5	DC	OL	29	Very high	High	Very high	YES
23	YES	77	Male	Ticagrelor	UFH	1	FNS	TL, BG, V	80	Low	High	Medium	YES
24	YES	66	Male	Clopidogrel	UFH	6	V	TL, OL	24	Very high	High	Very high	YES

UFH, unfractionated heparin; HA, headache; V, vomiting; DC, disturbance of consciousness; FNS, focal neurological signs; CH, cerebral; FL, frontal lobe; PL, parietal lobe; TL, temporal lobe; OL, occipital lobe; SS, subarachnoid space; BG, basal ganglia; CE, cerebellum; V, ventricle.

**Table 5:** ROC: predictive outcomes with the 3 risk scores.

Variables	AUC (95% CI)	*P*-value	Sensitivity	Specificity
CRUSADE score	0.500 (0.261–0.739)	1.000	0.692	0.364
ARC-HBR score	0.619 (0.388–0.849)	0.325	0.692	0.545
AUCITY score	0.545 (0.305–0.786)	0.706	0.846	0.364

ROC, receiver operating characteristics.

## 4 Discussion

Although rare, complications of ICH after PCI in patients with coronary artery disease have a poor prognosis and high mortality. In this single-center retrospective study, 24 patients were finally included for analysis. Of these, our findings were as follows: (1) More than half of the patients (*n* = 13) with concomitant ICH after PCI had a poor prognosis, especially those with an ICH volume of more than 30 cm^3^ and those who were on a ventilator during their hospital stay. (2) Of the 9 patients with ICH who occurred ventricular extension, a total of 6 (66.7%) died. (4) The CRUSADE, ACUITY, and ARC-HBR scores can complement each other in assessing the risk of ICH occurring after PCI.

Studies on ICH after PCI are scarce so far, especially during hospitalization. Yang et al. studied 121,066 patients undergoing PCI between 2013 and 2022 in the Fu Wai Hospital; they found that the incidence of ICH was 0.015%, and the 90-day mortality was very high (72.2%) (19). Myint et al. analyzed 560,439 patients undergoing PCI between 2007 and 2012 in the British Cardiovascular Intervention Society (BCIS) database and found that the incidence of ICH after PCI during hospitalization was 0.02% (20). Our study ultimately included 24 patients with ICH after PCI, with more than half of them (54.2%) occurring in-hospital death. There were some studies of ICH complicating PCI during postoperative follow-up. In one study that included 11,136 patients, 30 (0.27%) patients developed ICH in the first year after PCI (21). Furthermore, a study using the Korean National Health Insurance Service database found that the cumulative incidence of ICH was 0.54% at 1 year after PCI and increased relatively steadily by 0.25–0.30% per year thereafter (6).

The incidence of ICH after PCI is relatively rare but may result in life­changing disabilities or even death, so the prevention and treatment of ICH as well as the timely identification of high-risk groups are crucial, but the information of previous studies is limited, so more clinical studies are needed to provide support. Several studies have found advanced age, hypertension, and a history of stroke or transient ischemic attack to be independent predictors of ICH after PCI (6, 21). In our analysis, we found that more than half of ICH appeared within 12 h after PCI and early symptoms of impaired consciousness. Jeffrey et al. found that all hemorrhagic strokes occurred within 48 h of PCI in a study that included 5,372 patients with AMI treated with PCI, suggesting that the risk of early ICH after PCI is high (22). Awareness of complications of ICH is crucial in the early stages of patients undergoing PCI, especially in patients at high risk of bleeding. Therefore, clinicians should appropriately and comprehensively evaluate post-PCI patients for early identification of those at high risk of bleeding and provide aggressive symptomatic management.

One study found that clopidogrel and P2Y12 inhibitors were associated with a similar risk of ICH, which is consistent with our findings (23). The “Bleeding Academic Research Consortium” has put forward a standardized definition for post-PCI bleeding, with ICH being defined as a Type 3C bleed (24). The risk of ICH associated with DAPT is related to the individual and total potency of the drug. In the Stent Anticoagulation Restenosis Study, the risk of hemorrhagic complications in patients using aspirin, aspirin-ticlopidine, and aspirin-warfarin was 1.8%, 5.5%, and 6.2%, respectively (25). The above findings suggest that dual antiplatelet therapy in the perioperative period of PCI and the use of anticoagulant medications may be high-risk factors for the development of ICH, thus necessitating a thorough evaluation of antithrombotic strategies. In our study, aspirin combined with clopidogrel was used preoperatively in 45.8% of patients, and aspirin combined with ticagrelor in 54.2%.

As previously reported by Tuhrim et al., the 30-day mortality rate in patients with intracranial hemorrhage (ICH) was significantly higher when ventricular extension was present (26). Consistent with this, our data showed a high mortality rate among patients with ventricular extension. Specifically, among the 9 patients with ICH who experienced ventricular extension, a substantial proportion succumbed to their illness. Further investigation is warranted to explore the underlying mechanisms and potential interventions to improve outcomes in such high-risk cases. Therefore, it is necessary to review the head CT after the occurrence of ICH to detect any erroneous ventricular extension. Besides, we found that among the 24 patients included, 15(62.5%) patients had hypertension. A systematic review that enrolled 14 studies that examined the relationship between hypertension and ICH showed a positive correlation between hypertension and ICH (27). In addition, a multicenter, randomized phase III trial (ATACH II) demonstrated that aggressive control of blood pressure (target SBP level <140 mmHg) within 3 h of the onset of ICH reduced the risk of death or disability in the 3 months following ICH (28). Therefore, effective management of blood pressure might reduce the risk of bleeding after PCI. The American Heart Association guidelines on the management of ICH, recommend maintaining blood pressure levels below a mean arterial pressure of 130 mmHg (29).

In recent years, several risk scores have been utilized to evaluate bleeding in patients with coronary artery disease (CAD), including the CRUSADE score, ARC-HBR score, and ACUITY score. These scores have been widely applied to assess the risk of nosocomial bleeding in patients with CAD (30). For instance, Costa et al. found that in the overall patient population undergoing PCI, the CRUSADE score predicted major bleeding similarly to ACUITY (16). However, it should be noted that none of these scores have been specifically validated for predicting ICH after PCI. In our study, we explored the potential of these scores in assessing the risk of bleeding after PCI. Although we observed that the ARC-HBR score may have a slight advantage over CRUSADE and ACUITY in predicting poor ICH prognosis, this finding should be interpreted with caution due to the limited power of our study (30). Further large-scale, multi-center studies are needed to comprehensively evaluate the predictive ability of these scores for ICH after PCI.

In addition, we did not collect data on the proportion of patients with intracranial hemorrhage (ICH) and concomitant recent ischemic stroke after percutaneous coronary intervention (PCI), nor did we gather information on other potential causes of ICH. Future studies should consider collecting data on these aspects to provide a more comprehensive understanding of the clinical characteristics and outcomes of ICH after PCI. And the current study mainly focuses on the overall clinical characteristics and outcomes of hospitalized patients with intracranial hemorrhage (ICH) after percutaneous coronary intervention, without further subgroup division for survivors and non—survivors. Future studies may consider conducting subgroup analyses to further explore the differences and potential influencing factors in hospital stay days among patients with different characteristics.

In summary, because of the rapid deterioration, high mortality, and high healthcare costs once a patient undergoes ICH after PCI, it is critical to provide more clinical information to identify those at high risk of bleeding as early as possible and to help determine treatment strategies and clinical decisions. In addition, for the management of ICH after PCI, we recommend close neuromonitoring and early intervention to prevent sustained ICH extension while avoiding cardiovascular events during the temporary interruption of DAPT.

## 5 Limitations

Our study, as a single-center study, has several limitations that may affect the outcome and analysis. Firstly, this is a retrospective study and, therefore, suffers from the inherent limitations of observational databases. Secondly, out-of-hospital and asymptomatic ICH were not included in our study. Besides, our study did not routinely perform CT scans in patients after PCI; therefore, the incidence of subclinical ICH may have been underestimated. Thirdly, our study did not routinely explore the etiology of patients with ICH, and it is difficult to determine the cause of ICH because only an initial CT scan of the brain was performed without further imaging. Finally, there were some missing patient history data when collected in this study, which may affect the overall reliability of the analysis. Therefore, future large-scale, multicenter, and prospective studies are necessary to provide more evidence. Despite its limitations, it also has essential strengths worth considering. This study provides clinical practitioners with additional clinical evidence regarding the management of bleeding related to PCI, which can help to improve the prognosis of these patients.

## 6 Conclusions

The mortality of ICH after PCI was high and high volume of ICH indicates a high risk of death. Although ICH post-PCI is a rare, it remains a great challenge and dilemma regarding how to manage these patients. In our study, we provide clinical information on the clinical features, imaging manifestations, and mortality of ICH patients after PCI during hospitalization, providing assistance in developing optimal treatments to improve the prognosis of these patients.

## Data Availability

The raw data supporting the conclusions of this article will be made available by the authors, without undue reservation.
